# Evaluating stakeholder engagement in collaborative research: co-producing knowledge for climate resilience

**DOI:** 10.1007/s42532-022-00124-8

**Published:** 2022-08-22

**Authors:** Loretta Singletary, Elizabeth Koebele, William Evans, Christopher J. Copp, Shelby Hockaday, Jesse Jo Rego

**Affiliations:** 1 Departments of Economics and Cooperative Extension, University of Nevada Reno, Reno, NV USA; 2 Department of Political Science, University of Nevada Reno, Reno, NV USA; 3 Department of Human Development, Family Science, and Counseling, University of Nevada Reno, Reno, NV USA; 4 Interdisciplinary Doctoral Program in Social Psychology, University of Nevada Reno, Reno, NV USA; 5 Independent Science Researcher, Reno, NV USA; 6 Graduate Program in Hydrologic Sciences, University of Nevada Reno, Reno, NV USA

**Keywords:** Stakeholder engagement, Collaborative research, Knowledge co-production, Climate resilience, Food security, Snow-dependent river basins, Formative evaluation

## Abstract

This study describes the development, implementation, and evaluation of an initial stakeholder engagement experience designed to facilitate knowledge co-production. The engagement experience is part of a collaborative research framework (CRF), which facilitates iterative interactions among diverse researchers and stakeholders around the topic of enhanced climate resilience. Here, we describe the: (1) need for and development of a CRF as it relates to stakeholder engagement and knowledge co-production; (2) implementation of the initial engagement experience, focused around individual semi-structured interviews, in the context of a snow-dependent, arid river basin where historical water over allocation, climate change, and diversified water uses challenge the basin’s resilience; and (3) formative evaluation of the engagement experience using an online survey to inform the development of more effective engagement practices. Results of the evaluation indicate that, after participating, most stakeholders understand and recognize the importance of research goals, demonstrate positive attitudes toward collaborative research and researchers, view their contribution of knowledge and expertise as critical to research, and perceive researchers as eager to use their expertise. Moreover, stakeholders emphasized various context-specific goals for knowledge co-production, such as finding innovative ways to adapt to increased competition for diminishing water supplies. To achieve these goals, stakeholders suggested researchers learn about their basin, including its water allocation history and agricultural practices. These results highlight the importance of centering stakeholder engagement experiences within a broader CRF and formatively evaluating such experiences to adapt them to achieve research goals.

## Introduction

Despite increases in funding for scientific research on climate change impacts, mitigation, and adaptation, challenges remain for closing the gap between science research products and the public’s use of those products to achieve outcomes (Raaphorst et al. 2020; Cooke et al. 2021; Fischer et al. 2021). While scientists often perceive that they generate information intended to improve resource management decisions, resource managers often wonder why science does not provide the information they need to make decisions (Cooke 2019). Past reliance on top down, unidirectional science research that segregates academic disciplines and the research process from public involvement (Steelman et al. 2021) has inadvertently led to what has been called the science–policy divide (Steelman et al. 2019; Newcomb et al. 2021), knowledge–action gap (Knutti 2019), or theory–practice gap (Cooke et al. 2021). To help remedy this problem, transdisciplinary collaborative science research approaches have emerged to intentionally engage decision-makers and other stakeholders in the research process (Dekker et al. 2021; Steelman et al. 2021) with the goal of co-producing new knowledge that can inform and support actionable change on the ground (Caniglia et al. 2021). Ultimately, because neither scientists nor decision-makers alone can solve the kinds of complex resource management problems that climate change presents, collaboration between these groups is necessary (Vincent et al. 2018).

Evidence to date suggests that by engaging stakeholders in its production, scientific research becomes more transparent and legitimate to stakeholders (Singletary and Sterle 2020; Djenontin and Meadow 2018). In contributing to research, stakeholders acquire a sense of ownership of the research processes and outcomes (Norström et al. 2020). The resulting co-produced knowledge is perceived to be more useful at a relevant place-based scale, easier to integrate within an existing decision framework, and thus more likely to be used to make decisions (Dilling and Lemos 2011; Lemos et al. 2019; Tobias et al. 2019). Moreover, information exchange between scientists and stakeholders facilitates social learning and can identify areas of common ground in multi-party natural resource disputes (Singletary and Sterle 2018). Stakeholder engagement toward these ends can occur at one or multiple phases of research, including during the development of the research design, model specification, data collection, data analysis, and validation and distribution of research outcomes (Bremer and Meisch 2017).

While there are many documented benefits of engaging stakeholders in scientific research, such collaborations not only demand substantive time and resources to undertake, but little is known about how to maximize their effectiveness. Consequently, examples of best practices and metrics for empirically assessing what constitutes effective stakeholder engagement are evolving (Cronan et al. 2022; Harvey et al. 2019; Durose et al. 2018; Rigolot 2020). Robust systematic knowledge about engagement processes and outcomes is needed so that funding agencies, stakeholders, and researchers avoid wasting resources and potentially damaging relationships crucial to managing complex socio-environmental problems (Eaton et al. 2022, 2021). Recent analyses of stakeholder engagement in collaborative research suggest that key factors underlying success include researchers having a clear understanding of who, why, when, and how to engage (Muhar and Penker 2018, p. 6)-factors that should be determined by the research question(s), political context of the research problem, and the available time, resources, and capacities of the science team (Kliskey et al. 2021; Harvey et al. 2019; Klink et al. 2017). Additionally, while iterative engagement is thought to increase knowledge co-production and science utility (Lemos and Morehouse 2005), the optimal number of iterations, or engagement modality, remains less well understood (Eaton et al. 2021; Church et al. 2021; Bremer et al. 2019), and evaluations of outcomes such as increased adaptive capacity remain mixed (Mach et al. 2020; Church et al. 2022). In fact, the added time, resources, and skills required for engagement have been cited as an obstacle to the broader use of collaborative research, along with warnings of engagement fatigue and burnout for scientists and stakeholders alike (Dilling and Berggren 2015; Roux et al. 2021).

To advance empirical research on best practices for stakeholder engagement in collaborative research toward knowledge co-production, this paper outlines a collaborative research framework (CRF) grounded in Reed et al.’s (2018, pp. 13–18) theory of participation. We describe the initial implementation of the stakeholder engagement portion of our CRF in the Walker River Basin, California-Nevada, USA, as part of a project funded by the U.S. Department of Agriculture entitled Synthesizing kNowledge to Optimize Water Policy for Agriculture under Changing Snowpack (SNOWPACS),^1^ which centered on individual, semi-structured interviews with diverse stakeholders. We formatively evaluate the engagement experience through an online survey assessing how stakeholders perceived the engagement experience. Formatively evaluating and adapting engagement practices can improve the likelihood of knowledge co-production (Louder et al. 2021; Mach et al. 2020; Patton 2017) and help to ensure that engagement is structured at optimal frequency, duration, and modality at pivotal research stages (Louder et al. 2021; Dekker et al. 2021). The survey results reported here help us better understand what constitutes an effective engagement process and how such processes affect collaborative research outcomes. Thus, they can be used to adapt and improve the collaborative research process, especially when coupled with other formative evaluation mechanisms built into the CRF.

## Designing a collaborative research framework (CRF) for SNOWPACS

The SNOWPACS project aims to co-produce new knowledge to enhance climate resilience by supporting the adaptation of irrigated agricultural communities in the arid western USA to shifts in the timing and quantities of snowmelt-derived water supplies. Many western river basins are over-allocated, meaning more water has been granted to users through a water rights-based system than is available in most years, even when snowpack is 100% or more of its expected accumulation (Libecap 2011; Lee et al. 2020). Climate change, which reduces snowpack accumulation and impacts the timing and amount of annual runoff, is leading to more variable annual water supplies in this region (Dettinger et al. 2015; Harpold et al. 2017; Li et al. 2017; U.S. Global Climate Research Program 2018). This, in turn, worsens challenges for—and potentially increases competition among—a diversity of water users and other stakeholders. The SNOWPACS team includes researchers from multiple academic disciplines with expertise in hydrology, agricultural and resource economics, institutional analyses, environmental policy, collaborative research, informatics, and social psychology.

In this project, a subset of the SNOWPACS team (referred to as the CRF sub-team going forward) was tasked with designing and implementing a framework for collaboration among: (1) the multi-disciplinary members of the research team; and (2) stakeholders in selected “case study” river basins who represent diverse, competing water uses. The overarching goal of our CRF is to better understand the complex interrelationships (Prokopy et al. 2017; Wall et al. 2017; Gober 2018; Lemos et al. 2018) between climate-driven changes in mountain snowpack, downstream water availability, and water management decisions across water use sectors that characterize snow-dependent river basins. To achieve this, our CRF adopts a hybrid “top-down” and “bottom-up” approach to knowledge co-production (Reed et al. 2018, p. 5). The top-down portion involves researchers from multiple disciplines working together to identify a research problem and research questions prior to stakeholder engagement. This portion of the CRF is evaluated through an annual researcher online survey and biennial researcher interviews. The bottom-up portion of the CRF involves stakeholder engagement in the research process that is designed to help answer the questions posed in the top-down portion. As will be illustrated below, our initial engagement experience for this portion of the CRF involved conducting semi-structured interviews with diverse stakeholders to elicit input and feedback to inform the team’s scientific research and future stakeholder engagement. It was evaluated through an online survey in which stakeholders were asked about their engagement experience conducted by hired professional evaluators.

The design of the bottom-up portion of the CRF was guided by Reed et al. (2018), which asserts that the likelihood of achieving successful collaborative research outcomes can be increased by applying a theory of participation. Specifically, context, process design, power dynamics management, and scalar fit are each expected to contribute to explaining the outcomes of stakeholder engagement in collaborative research, especially in the context of natural resource management decisions. In terms of *context*, the existing participation culture, or the degree to which stakeholders have previously been engaged in research or desire to be engaged, influences the likelihood of successful engagement toward co-production. For example, collaborative efforts that involve stakeholders with prior collaborative experience have been shown to be more likely to lead to learning (Koebele 2019) and, ideally, knowledge co-production. As for process *design*, carefully, consistently structured stakeholder engagement processes with appropriate stakeholder representation may lead to more beneficial outcomes, as opposed to ad hoc engagement (Trachtenberg and Focht 2005).

The quality of stakeholder engagement is additionally influenced by *power dynamics* that make up stakeholders’ values and world views and influence how they construct and validate diverse types of knowledge (Whitton and Carmichael 2022). Failure to recognize and attend to such dynamics has been one of the leading factors underlying engagement failures (Turnhout et al. 2020). Power dynamics can be managed through process designs that recognize and value diverse stakeholder knowledges and epistemologies. That is, engagement experiences must be structured to ensure that representative stakeholders each have equal opportunities to contribute. Finally, engagement outcomes are highly *scalar-dependent* in terms of time and space. Institutional expectations, combined with time constraints imposed upon grant-funded initiatives (Newcomb et al. 2021), may limit the development and/or sustainability of long-term relationships between researchers and stakeholders (Church et al. 2022), further confounding collaborative research processes and outcomes (Worosz et al. 2022). Therefore, collaborative research designs should strive to match engagement modality and intensity to the goals of the research, recognizing that while stakeholders’ deeply held values change slowly, their preferences for policy solutions may be influenced over shorter timescales through social learning (Vincent et al. 2021, 2018; Slater and Robinson 2020; Gerlak et al. 2019; Djenontin and Meadow 2018) and deliberative knowledge exchange (Koebele 2020; Meadow et al. 2015). Similarly, engagement must be organized and implemented at a spatial scale relevant to the research problems and ecological system where decision-making authority occurs (Wyborn and Bixler 2013).

We incorporated these theoretical recommendations into our initial engagement experience by following Reed et al.’s (2018, p. 8) recommended knowledge co-production engagement practices, which include the following: (1) taking time to develop a full understanding of the study area to select and adapt as needed our engagement approach; (2) involving all affected parties early on to develop shared goals targeting outcomes based on relevant knowledge; (3) designing equal opportunities for stakeholders to participate and valuing all participants’ contributions; (4) coordinating engagement frequency and duration to match project progress toward its goals over time; and (5) ensuring stakeholders’ interests and decision-making authority are represented in terms of the spatial scale of the research questions being pursued. The following section describes how the CRF sub-team implemented the initial stakeholder engagement experience in one of the case study basins selected for the SNOWPACS project, followed by the results of the formative evaluation of the experience and lessons for knowledge co-production more broadly.

## Implementing the CRF in SNOWPACS

### Pre-engagement research activities: the study profile and stakeholder analysis

**Fig. 1:**
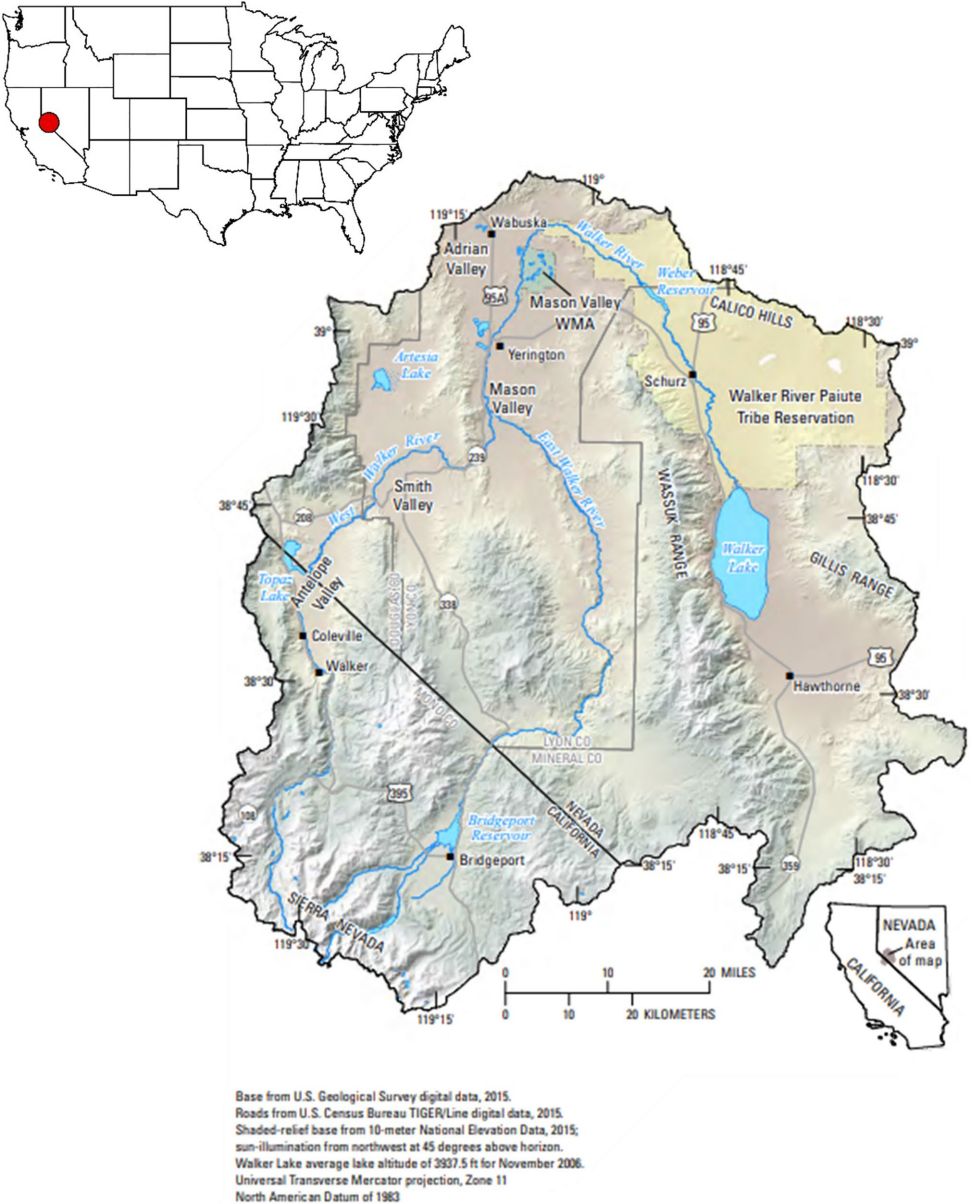
Map of Walker River Basin in Nevada, USA. Source: U.S. Geological Survey, 2022, Main Hydrologic Features in the Walker River Basin, accessed May 9, 2022 at URL https://nevada.usgs.gov/walker/walker.pdf. Inset map created in ArcGIS Pro by authors

The bottom-up portion of the CRF requires completing two steps prior to engaging stakeholders directly. First, the CRF sub-team developed a profile of the case study basin, the Walker River Basin (see Fig. 1), in which the initial engagement would occur, summarizing its geographic, economic, demographic, and institutional characteristics. The sub-team analyzed gray literature, including hydrologic, climate, and economic research conducted at the basin scale (Singletary et al. 2002; Singletary and Narayanan 2003; Carroll et al. 2010; Begay 2018), as well as archived documents (Horton 1996) that chronicle the basin’s water management over time. These documents helped the researchers understand the core water conflicts in the basin, which largely concern negative environmental impacts of historical agricultural water use on the basin’s terminus lake and wildlife (Wilds 2014).

Second, the CRF sub-team identified key actors across all water management sectors in the Walker River Basin as potential stakeholders to engage in collaborative research. We initially identified all stakeholders who could represent and contribute institutional knowledge, in addition to diverse values and interests, concerning water use and management; possessed power and/or authority to influence water management decisions; and could benefit from the process and outcomes of knowledge co-production (Prell et al. 2009; Reed et al. 2009, 2014, 2018; Reed and Curzon 2015). Once we identified prospective stakeholders, we selected individuals to participate who: (1) consume, deliver, protect, or supply water for primarily agricultural and environmental uses; (2) regulate water management decisions and policy; and (3) possess technical expertise and institutional knowledge concerning local water resource management practices and operations (Singletary and Sterle 2018). Ultimately, the selected individuals represented the diversity of water users in the basin, including irrigation districts and mutual water companies, environmental non-governmental organizations, federal and state water use regulatory bodies, and individual agricultural producers including those on tribal lands (Koebele et al. 2021).

### The engagement experience: individual interviews for knowledge co-production

The CRF sub-team initially engaged selected stakeholders through one-hour, semi-structured, individual interviews (Rubin and Rubin 2011). The purpose of the interviews was to work toward knowledge co-production by first gaining a better understanding of the study area and its water management challenges as perceived by diverse stakeholders and to discuss both actual and hypothetical adaptations to practices and policy that could facilitate climate change resilience. The CRF sub-team collaborated with other SNOWPACS researchers to get their input in developing interview question items, with the goal that stakeholder responses would inform broader climate–hydrologic–economic modeling efforts (Singletary and Sterle 2018).

We chose to conduct individual interviews, rather than utilizing focus groups or another form of direct stakeholder-to-stakeholder engagement, to address real or perceived power disparities among participants and encourage them to be frank in their responses. This is particularly important in our implementation of the CRF in the Walker Basin, given the high level of conflict among stakeholder groups. Furthermore, we promoted transparency and consistency by asking all interviewees the same set of questions, while also allowing for researcher discretion in prompting and following up with the interviewee on points of interest and clarification.

Stakeholders were invited to participate in interviews via an email invitation that included a one-page description of the research, as well as a link to a website where participants could learn more about the project goals and researchers prior to taking part. Due to the SARS-COVID-19 pandemic and university public health safety restrictions prohibiting in-person research in field settings, we conducted 28 interviews using Zoom video conferencing software, and three by telephone. Once university restrictions were reduced, we conducted two in-person interviews, following required social distancing procedures. The authors’ institutional review board reviewed and approved all primary data collection protocols and instrumentation associated with the interviews, which included an informed consent process. All interviews were recorded, transcribed, and qualitatively coded for future analyses.

During these interviews, the CRF sub-team: (1) introduced the SNOWPACS project based on the one-page description of the research that had been sent to stakeholders and (2) asked interview questions about stakeholders’ roles in the basin, types of water management challenges and decisions they make, what kinds of information water managers in the basin need, what coping actions they were taking in response to changing supply and demand, and similar topics. We also invited stakeholders to ask any questions they might have about SNOWPACS research goals and asked if they would be willing to continue engaging with researchers to inform the development of climate–hydrologic–economic models and verify model results. This question was intended to help stakeholders understand that the interviews were only a first step in a longer-term engagement with researchers and to communicate the importance of their knowledge to the SNOWPACS research.

This engagement experience served multiple purposes under the umbrella of the CRF. First, developing the interview questionnaire facilitated collaboration among the broader research team and helped connect scientists, beyond those working directly with stakeholders, to the engagement portion of the project (top-down portion). Second, the interview experience (bottom-up portion) provided foundational engagement between project researchers and basin stakeholders, informed stakeholders about the project goals and objectives, set the stage for on-going stakeholder participation, and allowed for baseline evaluation of stakeholder engagement experiences (Singletary and Sterle 2020), which will be discussed next. Third, project researchers are using the primary data collected during the interviews to analyze the potential for local institutional arrangements that might enhance and/or sustain water security in increasingly variable water supply conditions, as well as to help specify and test climate, hydrologic, and economic models being developed for the basin to simulate hypothetical adaptation strategies. These analyses represent co-produced knowledge that could not have been developed without diverse stakeholder input. Fourth, the CRF sub-team has summarized the data collected through stakeholder interviews for the broader SNOWPACS team and is preparing a series of Cooperative Extension publications to share research progress and findings with stakeholders and the broader public (see Singletary et al. 2022, for example). These publications are available to the public free of charge via the internet and public library access, and they are formatted following guidelines in the American Disabilities Act (1990) to increase information access diversity and inclusivity (Gould et al. 2019).

## Formatively evaluating the engagement experience

To better understand the effectiveness of our initial engagement experience, including how it might be adapted to better facilitate knowledge co-production goals and collaborative research broadly, we conducted a formative evaluation. To do this, after concluding each interview, the interviewer asked each stakeholder if they would be willing to take part in a short online survey designed to evaluate their engagement experience with the SNOWPACS project and CRF sub-team. Stakeholders who indicated willingness were sent a link to a Qualtrics survey within 24–48 h of the interview in an email thanking them for their participation. While all 33 interviewees agreed to complete an evaluation, only 73% (n = 24) completed the survey.

The survey questions were developed to assess the stakeholder engagement experience based on recommended practices grounded in participation theory (Reed et al. 2018), described above. Specifically, we used closed ended questions to measure stakeholders’: (1) understanding of project goals; (2) attitudes toward collaborating in SNOWPACS research; (3) past collaborative research engagement experiences; (4) attitudes toward SNOWPACS researchers; (5) perceived importance of their contributions influencing project success; (6) perceived value of how their individual expertise contributes to SNOWPACS research; (7) expectations for the project’s outcomes; (8) confidence that SNOWPACS researchers will use the knowledge stakeholders contribute; (9) perceived importance of the project goals; and (10) satisfaction with existing water allocation and projection models for their area. These questions were all measured using a 7-point Likert-type scale, where 1 = strongly disagree, 4 = neither agree nor disagree, and 7 = strongly agree, except for the last question which was measured on a reverse scale as an attention check for respondents, where 1 = extremely satisfied and 7 = extremely dissatisfied. Descriptive statistics for the closed-ended questions were calculated using Qualtrics.

Additionally, the online survey included three open-ended questions asking stakeholders to: (1) identify the largest water related concern in their area; (2) describe how they would define success for this project; and (3) explain what they believed researchers should know about their local area to inform this research. The qualitative responses were analyzed using thematic content analysis (Auerbach and Silverstein 2003; Glaser and Strauss 1999), in which the authors familiarized themselves with the overall data and inductively developed the key themes based on the questions and responses. To bring stakeholders’ voices to the fore, we include select stakeholder quotations below when presenting the results of this analysis.

### Formative evaluation results: closed-ended questions

Table 1 reports the mean scores and standard deviations for the 10 closed-ended questions. Results show that most respondents perceived the initial engagement experience positively. Indeed, 96% of participating stakeholders agreed that they understand the SNOWPACS project goals (M = 5.88; SD = 0.68) and looked forward to working with researchers on the project (M = 6.13; SD = 0.74).

**Table 1:** Stakeholder survey mean scores and standard deviations

Survey Item	Mean	SD
I understand the goals of the SNOWPACS project	5.88	0.68
I am looking forward to working with researchers on the SNOWPACS project	6.13	0.74
Stakeholders will be critical to the success of the SNOWPACS project	6.39	0.89
I have worked on similar projects in the past	4.78	1.44
SNOWPACS researchers are eager to incorporate my knowledge	5.96	0.82
I feel like I have a lot to offer this project in terms of my expertise	5.48	0.79
I am not personally expecting to get anything out of this project	4.57	1.83
I believe my knowledge will be used by SNOWPACS researchers	5.96	0.56
It is important that I know the goals of the SNOWPACS project	5.83	0.83
How satisfied are you with the current water allocation models and water projections for the work you do?	2.64^**a**^	1.23^**a**^

^a^This question, which does not specifically inquire about the engagement experience, was evaluated on a different 7-point Likert-type scale that was reverse coded, where 1 = extremely satisfied, 4 = neither satisfied nor dissatisfied, and 7 = extremely dissatisfied.

Additionally, most respondents reported having prior experience participating in research projects like SNOWPACS, with 65% indicating some level agreement on this question (M = 4.78; SD = 1.44), although the standard deviation is large, suggesting highly varied responses for this question across our sample. Stakeholders perceived SNOWPACS researchers as eager to incorporate their knowledge into project research (M = 5.96; SD = 0.082), with 91% of respondents responding positively, while the remaining 9% of participants neither agreed nor disagreed. From these results, we glean that we selected a set of stakeholders with fairly high levels of engagement experience through our stakeholder analysis, and the respondents believe that researchers are interested in what they have to say. Additionally, most stakeholders think their participation will be critical to the success of SNOWPACS (M = 6.39; SD = 0.89), and the average respondent also believed they have a lot of expertise to offer this research (M = 5.48; SD = 0.79), with 39% agreeing with this statement and 43% somewhat agreeing. These results suggest that stakeholders see value in engaging in collaborative research, making this project more likely to lead to knowledge co-production.

Respondents were most mixed on the question item, “I am not personally expecting to get anything out of this project,” (M = 4.57; SD = 1.83), as this relatively large standard deviation reveals, with 26% of participants agreeing with this statement and 17% of participants disagreeing. That said, respondents generally agreed that their knowledge would be used by researchers (M = 5.96; SD = 0.56). Respondents also believed it was important that they know the goals of SNOWPACS (M = 5.83; SD = 0.83). These results suggest that while stakeholders may see their knowledge and input as valuable to scientific research projects that they understand, they may not necessarily benefit personally from this research, though perhaps their broader stakeholder community might.

Finally, 62% of the participants reported only moderate satisfaction with the basin’s existing water allocation models and water supply projections (M = 2.64; SD = 1.23), with 8% of participants reporting moderate dissatisfaction, 24% reporting being slightly satisfied, and 6% reporting being neither satisfied nor dissatisfied on this question. This tepid satisfaction with information available to guide water management decisions under changing snowpack and snowmelt timing suggests that stakeholders may be more open to investigating alternative management regimes and coping actions, which is a key goal of the SNOWPACS project.

### Formative evaluation results: open-ended questions

The first open-ended question asked stakeholders what the largest water-related challenges in their area were. Sixteen participants responded to this question. Most highlighted the increasing competition for available water supplies, which create imbalances and tensions within their local area. This includes the perception that existing water allocation institutions may be out of step with current water management challenges. As one stakeholder commented, “*Laws governing water resources are outdated and are not based on science or the hydrology of the basin*.” Stakeholders also reported that climate change and inconsistent weather patterns presented new challenges to their existing water management responsibilities. One stakeholder summarized these challenges as follows: “*Changes in the amount and timing of precipitation are combining with increases in temperature and with continued population growth and residential development to exacerbate existing imbalances and tensions between the available supply of fresh water and the demands of urban, suburban, exurban and rural communities and the needs of the environment. Within the context of this increasingly sharp competition over limited available freshwater resources, poorer communities and the environment are in danger of losing out in ways that jeopardize their continued viability.”* Similar responses to this question suggest broadly that stakeholders acknowledge the potential for growing competition over increasingly limited water resources to increase conflict, further validating the need for knowledge that can aid stakeholders in finding both common ground and new solutions.

Second, we asked stakeholders how they would define success for a collaborative research and water modeling project such as SNOWPACS. Seventeen participants responded, with most defining project success as improved management practices or new information that could inform such efforts. Comments included, for example, “*Allowing for additional forecasting that may help with water acquisition strategies for instream use (price, type of water)*” and “*Water users, administrators, and law makers are able to rely on the final product to improve how water is managed.”* Stakeholders also described project success in terms of help in dealing with repercussions from climate change already being felt. Such comments included, for example, “*Realistic and implementable strategies by water users to adapt to changing water supply” and “The development of a tool or model that operators can use to make seasonable [sic] decisions for their operations.”* Some participants encouraged researchers to aim for project outcomes that address larger social welfare concerns including, for example, “*Models need to include the effect that government agency regulations have on a watershed and not just climate, streamflow, and water management,”* and “*A resource [is needed] that builds consensus and understanding*.” As one stakeholder commented, “*Success would be represented by water modeling of reliable accuracy that factors in the needs of water-dependent natural resources and land-based rural communities, and that explores the ramifications of various alternative water resource management scenarios for meeting those needs*.” Together, these responses suggest that stakeholders need both procedural and substantive solutions to climate resilience challenges in their river basin.

Finally, we asked participants what researchers should know about their area to better inform project research. Responses to this item were quite diverse. Some participants suggested researchers might need to learn about local and state water laws and regulations that govern water use in the basin. As one stakeholder summarized, “*First they need to understand and study Nevada water law, then finding a balance for all users of water.*” Others suggested researchers learn about water cycles within their basin as well as other relevant basins. Comments included for example, [researchers need to learn] “*History of our waters and how we got to a point in time and stopped improving,*” and “*I think understanding water rotations in a given ditch system is very important to how water losses are shared*.” Stakeholders also encouraged researchers to learn about ways that local farmers were practicing sustainable agriculture, as well as the effect of agricultural diversions on other parts of the system: “*I think researchers need to know about and consider how to meet the need for additional flows of waters to restore and maintain a reasonable state of ecological health and function in Walker Lake and the riparian and estuarine habitat areas of the lower reach of the Walker River*.” Similarly, one stakeholder noted that [researchers need to] “*understand the efficiency and extent of aquifer recharge from mountain snowpack infiltration and runoff on a basin-by-basin scale—essentially the perennial groundwater yield of a basin and how the perennial yield relates to the system yield as in a stream-dominated system like the Walker*.” Ensuring researchers understand the constraints and opportunities for sustainable water management, whether physical or social, appears to be a common concern, regardless of water use sector.

## Implications for knowledge co-production processes

Research to date on climate science knowledge co-production suggests that success hinges in large part on the quality of collaborative interactions between researchers and stakeholders (Cundill et al. 2019). The frequency and duration of these interactions have been linked to the production of products with greater utility (Jacobi et al. 2022; Prokopy et al. 2015; Lemos et al. 2019). Yet, researchers and stakeholders alike continue to grapple with various challenges specific to collaborative climate research (Kirchhoff et al. 2013, 2015; Briley et al. 2015), which is often described as ambiguous and lacking in empirical or evidence-based strategies for implementation and metrics for monitoring its progress and outcomes (Eaton et al. 2021; Singletary and Sterle 2020; Wall et al. 2017).

Our study approaches this empirical gap through a formative evaluation of an engagement experience aimed to support the adaptation of agricultural communities in the western USA to shifts in the timing and quantities of snowmelt-derived water supplies. To incorporate concerns of context, process design, power dynamics management, participation culture, and scalar fit (Reed et al. 2018), the CRF sub-team first developed a profile of the study area, the Walker River Basin, which helped us to learn about the historical water allocation institutions unique to the basin of interest, understand past and current water supply and demand management challenges, and identify potential stakeholders to engage. We then combined the results of this profile with results of a stakeholder analysis to identify water managers across the Walker River Basin who could contribute diverse knowledge and perspectives surrounding water management challenges and potential solutions under a changing climate. To initially engage these stakeholders in knowledge co-production, we conducted individual in-depth, semi-structured interviews. We designed these interviews to inform SNOWPACS empirical research while also building relationships between researchers and stakeholders to set the stage for a longer-term collaborative research process (Bojovic et al. 2021). Finally, to assess the engagement experience, we evaluated stakeholders’ perceptions of it through an online survey.

Our survey results indicate that our CRF has been successful in helping to identify and engage stakeholders who possessed prior engagement experience, which has been shown to improve the likelihood of learning and knowledge co-production in other contexts (Koebele 2019; Norström et al. 2020). The results also show that our initial engagement experience communicated the project’s scope of work to stakeholders, who indicated that because of this initial engagement, they understand and recognize the importance of understanding the project’s goals. Further, stakeholders expressed positive attitudes toward collaborative research processes broadly and toward collaborating with SNOWPACS researchers specifically. Critically, they perceived that their individual expertise would be integrated into and contribute to successful outcomes from SNOWPACS. Together, these results suggest this type of structured engagement helps to create a safe, creative space for learning and knowledge exchange among researchers and stakeholders (Arnott et al. 2020).

Stakeholders reported only moderate satisfaction with existing water allocation models and water supply projections for the basin, with two participants reporting moderate dissatisfaction, which emphasizes stakeholders’ perceived need for new knowledge to inform and improve water management decisions in the basin. At the same time, stakeholders expressed an urgent need for the SNOWPACS project to co-produce new knowledge relevant to issues specific to the Walker River Basin, which speaks to concerns about the scalar fit of the research. For instance, stakeholders described increasing competition from the environmental water use sector to acquire agricultural water rights, compounded by inconsistent weather patterns, as substantive issues they face, which also reaffirms the need to manage power dynamics in co-production processes. Further, stakeholders recommended that SNOWPACS researchers should learn about their basin in terms of historical water cycles and to identify and provide information to help manage water at the farm level. This outcome of engagement reiterates and verifies the importance of both context and spatial consideration in the design of engagement processes (Reed et al. 2018). For these stakeholders, project success will mean potentially new water allocation or management policies at the basin level, in addition to more reliable predictions of annual water availability. In short, formative evaluation results revealed important, actionable knowledge gaps in this river basin, which SNOWPACS has an opportunity to fill through continued, structured, and responsive stakeholder engagement (Mach et al. 2020).

### Next steps

The formative evaluation results from the stakeholder engagement experience reported here are only one part of the bottom-up portion of the CRF. At the time of writing this manuscript, stakeholders who offered to participate in SNOWPACS on an on-going basis were recruited as technical advisors who will collaborate with researchers to inform model specification, refine model performance, and verify model outputs. These activities will further inform best practices for stakeholder engagement toward knowledge co-production by providing additional insight into how to best structure interactions between researchers and stakeholders. They also help to integrate the top-down and bottom-up portions of the CRF, as researchers beyond the CRF sub-team will interact directly with stakeholders. The CRF sub-team is also replicating this initial engagement experience in a second river basin, which will allow for a comparative assessment of engagement experiences across settings with different water challenges, geographic scales, demographics, economics, and institutions.

The top-down portion of the CRF additionally includes assessing SNOWPACS researchers’ attitudes toward collaborative research generally, and specifically toward the CRF designed and implemented for this project. Understanding researchers’ perceived challenges for collaborating across disciplines and with stakeholders will help to inform future research design and engagement practices.

### Study challenges and limitations

Project implementation coincided with the beginning of the SARS-COVID-19 pandemic, which introduced unexpected and unprecedented challenges for stakeholder engagement modalities planned for the project that originally had included multiple iterative in-person interactions. The authors’ University Office of Research Integrity imposed public health safety precautions that included several months of prohibited in-person field research. These unexpected restrictions required that we adapt our engagement, replacing planned in-person interviews with virtual video conference technology and telephone interactions.

While the pandemic continues to influence engagement modality, slowing the overall pace and texture of this research project, we have discovered that Walker River Basin stakeholders show participatory resilience. That is, despite the impact of the pandemic on their respective professional and personal lives, most of the stakeholders we have engaged consistently show a willingness to take part in this project’s knowledge co-production processes. The formative evaluation results presented here suggest that this willingness may in part represent a real need for new co-produced knowledge for adapting to climate change impacts, as well as an effective process design with products that could inform future local water management decisions.

Finally, the potential for response bias exists in the evaluation data reported here. Nine of the 33 stakeholders who did not complete the online survey may have been dissatisfied with their initial engagement experience. Subsequently, it is possible that the responses reported here may be positively skewed.

## Reflections on knowledge co-production going forward

Recent years of federally funded co-produced knowledge initiatives in the USA specific to climate change have prompted scientists and stakeholders alike to advocate for its widespread use and funding (Cundill et al. 2019; Lemos et al. 2019; Arnott et al. 2020). While transdisciplinary collaborative research for knowledge co-production is an increasingly common approach to closing the gap between climate science and action (Kirchhoff et al. 2013, 2015; Flagg and Kirchhoff 2018), questions remain concerning what constitutes effective co-production processes and outcomes (Howarth et al. 2022; Bremer and Meisch 2017). The formative evaluation results of the engagement experience reported here suggest that designing co-production processes and outcomes around core principles, as outlined in participation theory, can help to influence project success or failure. This necessarily includes creating opportunities that can help establish or enhance a culture of participation. Additionally, diverse modalities of engagement exist, wherein researchers and stakeholders co-engage one another across a range of complex resource problems and contexts. This suggests that effective knowledge co-production does not necessitate high frequency iterative stakeholder engagement, which may lead to engagement fatigue, but instead requires an organic approach to tailoring engagement experiences responsive to the nuances of the context *and* scope of work. Recognizing, seeking out, and embracing this level of engagement diversity can serve to advance collaborative research processes and impacts (Mach et al. 2020).

Defining what constitutes effective knowledge co-production and best stakeholder engagement practices continues to evolve, in part thanks to a growing international body of scholars pursuing these linkages (see Eaton et al. 2022). Research is needed, for example, to clarify how features of engagement processes influence social learning, capacity building, and behavioral changes that lead to environmental changes (Eaton et al. 2021, p. 1117). Also, while advocates for knowledge co-production and actionable science argue for its positive effects on building adaptive capacity, empirical work to assess causality, as well as the potential for negative outcomes from engagement, is needed (Eaton et al. 2021, p. 1126). To solidify the promise of knowledge co-production, a clear appraisal is required of the set of conditions from which stakeholder engagement and learning processes bring about positive social and environmental change (Gerlak et al. 2019). Without unpacking what Eaton et al. (2021) refer to as a ‘black box,’ “…we risk undertaking stakeholder engagement processes without clear knowledge of the type of change that may be obtainable, how change is catalyzed, and how we can causally link engagement processes and outcomes” (Eaton et al. 2021, p. 1112).

Federally sponsored, co-produced climate knowledge projects across the USA are reporting enhanced utility of that knowledge (e.g., Prokopy et al. 2017; Babin 2018; Singletary and Sterle 2020) and citing challenges–often referred to as ‘lessons learned’ (Hegger et al. 2012; Hegger and Dieperink 2014; Ferguson et al. 2017; Church et al. 2019). We recommend ongoing evaluation of these processes to help evolve evidence-based engagement practices. Such evaluative research can illuminate the engagement modalities and intensity most likely to facilitate co-creation of actionable science that supports climate resilience (Eaton et al. 2022; Louder et al. 2021; Kliskey et al. 2021; Arnott et al. 2020; Allen et al. 2017; Ferguson et al. 2018; Lemos et al. 2018). Consistently evaluating collaborative research processes and outcomes can also help to explore how stakeholder heterogeneity can inform and improve knowledge co-production processes. That is, different stakeholders operating at different scales of resource management and decision-making are likely to have different information needs and therefore may require very different modes of engagement (Kliskey et al. 2021; Reed et al. 2009). Information needs likely vary, for example, for individual producers making decisions at the farm scale; local and regional managers making decisions at a conservation or irrigation district or at a county scale; and policymakers making decisions at the basin, state, inter-state, multi-state, or national scale (Durose et al. 2017, 2018).

Finally, transdisciplinary collaborative research and knowledge co-production may not persevere without protecting researchers and stakeholders from their respective organizational culture, which may reward more conventional and narrowly focused endeavors. Protection may include combinations of moderating performance criteria that explicitly nurture or at least support a culture of knowledge co-production and participation (Boon et al. 2019), which in turn can help our communities become more resilient in the face of climate change.

### International implications for knowledge co-production

Knowledge co-production to enhance and support climate adaptation of agricultural communities across the western USA can inform similar undertakings in arid, snow-dependent riverine environments around the globe—and vice versa. Many agricultural water users worldwide share similar circumstances in that they face more variable water supplies (Qin et al. 2020) coincident with increasing competition for water from municipal entities, to support rapidly growing urban centers, and from environmental entities to ensure adequate water for wildlife habitat and ecosystem health. Furthermore, the quality of interactions among diverse, competing water use interests will affect important future decisions surrounding water reallocation and related goals, such as global food security. Highly structured and thoughtful engagement can strive to build and support the co-creation of information and relationship-building around common challenges, potentially enhancing adaptive capacity and resilience (Church et al. 2019, 2021).

Further, as collaborative research continues to bring together diverse perspectives and knowledges to address wicked problems of the twenty-first century (Chan and Xiang 2022; Wyborn et al. 2019; Lukasiewicz and Baldwin 2017), it is important to recognize that knowledge co-production means different things to different people and in different contexts (Zurba et al. 2022, p. 451). Having a set of proven principles and best practices to guide engagement experiences toward knowledge co-production will become even more important. However, in many places and for many people worldwide, such principles and practices may be insufficient to account for systemic and highly contextualized issues such as the effects of colonization and data sovereignty on Indigenous peoples and communities (Zurba et al. 2022, p. 450). Therefore, high-quality knowledge co-production processes aimed at long-term change must carefully attend to power dynamics management, including who should be considered for engagement (Gagnon et al. 2022, p. 11), as well as local context, as was done in this study.

## Data Availability

As this research involves interviewing and surveying human subjects, data will be stored following the University of Nevada, Reno Institutional Review Board guidelines, for which this research was approved.

## Footnotes

1 SNOWPACS website: https://packpages.unr.edu/snowcap/.

## References

[CR1] Allen E, Stephens J, Yorgey G, Kruger C, Ahamed S, Adam J (2017). Climate science information needs among natural resource decision-makers in the Northwest U.S. Clim Serv.

[CR2] Arnott JC, Neuenfeldt RJ, Lemos MC (2020). Co-producing science for sustainability: can funding change knowledge use?. Glob Environ Change.

[CR3] Auerbach C, Silverstein LB (2003). Qualitative data: an introduction to coding and analysis.

[CR4] Babin N (2018) NIFA water synthesis case study, water sustainability in snow-fed arid land river systems. USDA NIFA, West Lafayette, IN

[CR5] Begay M (2018) Walker River Paiute Tribe climate adaptation plan. http://paiutewater.us/wrpt_climate_change_plan_nov2018.pdf. Accessed 15 Nov 2021

[CR6] Bojovic D, St ClairChristelTerradoStanzel ALEMP, Gonzalez P, Palin EJ (2021). Engagement, involvement and empowerment: three realms of a coproduction framework for climate services. Glob Environ Change.

[CR7] Boon W, Hessels L, Horlings E (2019). Knowledge co-production in protective spaces: case studies of two climate adaptation projects. Reg Environ Change.

[CR8] Bremer S, Meisch S (2017). Co-production in climate change research: reviewing different perspectives. Wires Clim Change.

[CR9] Bremer S, Wardekker A, Dessai S, Sobolowski S, Slaattelid R, van der Sluijs J (2019). Toward a multi-faceted conception of co-production of climate services. Clim Serv.

[CR10] Briley L, Brown D, Kalafatis SE (2015). Overcoming barriers during the co-production of climate information for decision-making. Clim Risk Manag.

[CR11] Caniglia G, Luederitz C, von Wirth T, Fazey I, Martín-López B, Hondrila K, König A, von Wehrden H, Schäpke NA, Laubichler MD, Lang DJ (2021). A pluralistic and integrated approach to action-oriented knowledge for sustainability. Nat Sustain.

[CR12] Carroll RWH, Pohll G, McGraw D, Garner C, Knust A, Boyle D, Minor T, Bassett S, Pohlmann K (2010). Mason valley groundwater model: linking surface water and groundwater in the Walker River Basin, Nevada. J Am Water Resour Assoc.

[CR13] Chan JKH, Xiang WN (2022). Fifty years after the wicked-problems conception: its practical and theoretical impacts on planning and design. Socio-Ecol Pract Res.

[CR14] Church SP, Babin N, Bentlage B, Dunn M, Ulrich-Schad JD, Ranjan P, Magner J, McLellan E, Stephan S, Tomer MD, Prokopy LS (2019). The beargrass story: utilizing social science to evaluate and learn from the “watershed approach”. J Contemp Water Res Educ.

[CR15] Church SP, Floress KM, Ulrich-Schad JD, Wardropper CB, Ranjan P, Eaton WM, Gasteyer S, Rissman A (2021). How water quality improvement efforts influence urban–agricultural relationships. Agric Hum Values.

[CR16] Church SP, Wardropper CB, Usher E, Bean LF, Gilbert A, Eanes F, Ulrich-Schad JD, Babin N, Ranjan P, Getson JM, Esman LA, Prokopy LS (2022) How does co-produced research influence adaptive capacity? Lessons from a cross-case comparison. Socio Ecol Pract Res 4(3). 10.1007/s42532-022-00121-x

[CR17] Cooke SJ (2019). From frustration to fruition in applied conservation research and practice: ten revelations. Socio Ecol Pract Res.

[CR18] Cooke SJ, Jeanson AL, Bishop I, Bryan BA, Chen C, Cvitanovic C, Fen Y (2021). On the theory-practice gap in the environmental realm: perspectives from and for diverse environmental professionals. Socio-Ecol Pract Res.

[CR19] Cronan D, Trammell EJ, Kliskey A (2022). Images to evoke decision-making: building compelling representations for stakeholder-driven futures. Sustain.

[CR20] Cundill G, Harvey B, Tebboth M, Cochrane L, Currie-Alder B, Vincent K, Lawn J, Nicholls RJ, Scodanibbio L, Prakash A, New M, Wester P, Leone M, Morchain D, Ludi E, DeMaria-Kinney J, Khan A, Landry M-E (2019). Large-scale transdisciplinary collaboration for adaptation research: challenges and insights. Glob Chall.

[CR21] Dekker R, Geuijen K, Oliver C (2021). Tensions of evaluating innovation in a living lab: moving beyond actionable knowledge production. Evaluation.

[CR22] Dettinger M, Udall B, Georgakakos A (2015). Western water and climate change. Ecol Appl.

[CR23] Dilling L, Berggren J (2015). What do stakeholders need to manage for climate change and variability? a document-based analysis from three mountain states in the western USA. Reg Environ Change.

[CR24] Dilling L, Lemos MC (2011). Creating usable science: opportunities and constraints for climate knowledge use and their implications for science policy. Glob Environ Change.

[CR25] Djenontin INS, Meadow AM (2018). The art of co-production of knowledge in environmental sciences and management: lessons from international practice. Environ Manag.

[CR26] Durose C, Needham C, Mangan C, Rees J (2017). Generating 'good enough' evidence for co-production. Evid Policy.

[CR27] Durose C, Richardson L, Perry B (2018). Craft metrics to value co-production. Nature.

[CR28] Eaton W, Brasier KJ, Burbach M, Whitmer W, Engle EW, Burnham M, Quimby B, Chaudhary AK, Whitley H, Delozier J, Fowler LB, Wutich A, Bausch JC, Beresford M, Hinrichs CC, Burkhart-Kriesel C, Preisendanz HE, Williams C, Watson J, Weigle J (2021). A conceptual framework for social, behavioral, and environmental change through stakeholder engagement in water resource management. Soc and Nat Res.

[CR29] Eaton W, Robertson T, Arbuckle J, Brasier KJ, Burbach M, Burnham M, Church S, Eberly G, Hart-Fredeluces G, Jackson-Smith D, Rogers A, Wildermuth G, Canfield K, Cordova S, Chatelain C, Edwards J, Fowler L, Hurst Z, Kirchhoff C, Manheim M, Martinez R, Mook A, Mullin C, Murrah-Hanson L, Onabola C, Parker L, Redd E, Schelly C, Schoon M, Sigler W, Smit E, van Huysen T, Verbrugge L, Worosz M (2022) Advancing scholarship and practice of stakeholder engagement in working landscapes: 34 co-produced research opportunities. Engagement workshop series report. The Pennsylvania State University. https://scholarsphere.psu.edu/resources/d6066f7e-045c-41f7-af69-9cc15e1e81f3

[CR30] Ferguson L, Chan S, Santelmann M, Tilt B (2017). Exploring participant motivations and expectations in a researcher-stakeholder engagement process: willamette water 2100. Landsc Urban Plan.

[CR31] Ferguson L, Chan S, Santelmann M, Tilt B (2018). Transdisciplinary research in water sustainability: what's in it for an engaged researcher-stakeholder community?. Water Altern.

[CR32] Fischer LJ, Wernli H, Bresch DN (2021). Widening the common space to reduce the gap between climate science and decision-making in industry. Clim Serv.

[CR33] Flagg JA, Kirchhoff CJ (2018). Context matters: context-related drivers of and barriers to climate information use. Clim Risk Manag.

[CR34] Gagnon V, Schelly C, Lytle W, Kliskey A, Dale V, Marshall A, Rodriguez L, Williams P, Price M, Redd E, Noodin M (2022). Enacting boundaries or building bridges? language and engagement in food-energy-water systems science. Socio-Ecol Pract Res.

[CR35] Gerlak AK, Heikkila T, Smolinski SL, Armitage D, Huitema D, Moore B (2019). It’s time to learn about learning: where should the environmental and natural resource governance field go next?. Soc and Nat Res.

[CR36] Glaser BG, Strauss AL (1999). Discovery of grounded theory: strategies for qualitative research.

[CR37] Gober P (2018). Social learning for water sector resilience.

[CR38] Gould R, Harris SP, Mullin C (2019) Higher Education and the ADA: an ADA Knowledge Translation Center Research Brief. https://adata.org/research_brief/higher-education-and-ada

[CR39] Harpold A, Dettinger M, Rajagopal S (2017). Defining snow drought and why it matters. Eos.

[CR40] Harvey B, Cochrane L, van Epp M (2019). Charting knowledge co-production pathways in climate and development. Environ Policy Gov.

[CR41] Hegger D, Dieperink C (2014). Toward successful joint knowledge production for climate change adaptation: lessons from six regional projects in the Netherlands. Ecol Soc.

[CR42] Hegger D, Lamers M, van Zeijl-Rozema A, Dieperink C (2012). Conceptualising joint knowledge production in regional climate change adaptation projects: success conditions and levers for action. Environ Sci Policy.

[CR43] Horton G (1996) A chronological history of the Walker River and related water issues: a publication in the Nevada Water Basin information and chronology series. http://images.water.nv.gov/images/publications/River%20Chronologies/Walker%20River%20Chronology.pdf. Accessed 3 Dec 2021

[CR44] Howarth C, Lane M, Morse-Jones S, Brooks K, Viner D (2022). The ‘co’ in co-production of climate action: challenging boundaries within and between science, policy and practice. Global Environ Change.

[CR45] Jacobi J, Llanque A, Mukhovi SM, Birachi E, von Groote P, Eschen R, Hilber-Schöb I, Kiba DI, Frossard E, Robledo-Abad C (2022). Transdisciplinary co-creation increases the utilization of knowledge from sustainable development research. Environ Sci Policy.

[CR46] Kirchhoff CJ, Carmen Lemos M, Dessai S (2013). Actionable knowledge for environmental decision making: broadening the usability of climate science. Annu Rev Environ Resour.

[CR47] Kirchhoff CJ, Lemos MC, Kalafatis S (2015). Narrowing the gap between climate science and adaptation action: the role of boundary chains. Clim Risk Manag.

[CR48] Klink J, Koundinya V, Kies K, Robinson C, Rao A, Berezowitz C, Widhalm M, Prokopy L (2017). Enhancing interdisciplinary climate change work through comprehensive evaluation. Clim Risk Manag.

[CR49] Kliskey A, Williams P, Dale VH, Schelly C, Marshall A, Griffith D, Eaton W, Floress K, Gagnon V (2021). Thinking big and thinking small: a conceptual framework for best practices in community and stakeholder engagement in food, energy, and water systems. Sustain.

[CR50] Knutti R (2019). Closing the knowledge-action gap in climate change. One Earth.

[CR51] Koebele EA (2019). Policy learning in collaborative environmental governance processes. J Environ Policy Plan.

[CR52] Koebele EA (2020). Cross-coalition coordination in collaborative environmental governance processes. Policy Stud J.

[CR53] Koebele E, Singletary L, Hockaday S, Ormerod KJ (2021) What role can water markets play in adapting to climate change? evidence from two river basins in the western United States. In: Duerk JC (ed) Environmental philosophy, politics, and policy. Lexington Books, Lanham, MD

[CR54] Lee G-E, Rollins K, Singletary L (2020). The relationship between priority and value of irrigation water used with prior appropriation water rights. Land Econ.

[CR55] Lemos MC, Morehouse BJ (2005). The co-production of science and policy in integrated climate assessments. Glob Environ Change.

[CR56] Lemos MC, Arnott JC, Ardoin NM (2018). To co-produce or not to co-produce. Nat Sustain.

[CR57] Lemos M, Wolske K, Rasmussen L, Arnott J, Kalcic M, Kirchhoff C (2019). The closer, the better? Untangling scientist–practitioner engagement, interaction, and knowledge use. Weather Clim Soc.

[CR58] Li D, Wrzesien ML, Durand M, Adam J, Lettenmaier DP (2017). How much runoff originates as snow in the western United States, and how will that change in the future?. Geophys Res Lett.

[CR59] Libecap GD (2011). Institutional path dependence in climate adaptation: Coman's "some unsettled problems of irrigation". Am Econ Rev.

[CR60] Louder E, Wyborn C, Cvitanovic C, Bednarek AT (2021). A synthesis of frameworks available to guide evaluations of research impacts at the interface of environmental science, policy, and practice. Environ Sci Pol.

[CR61] Lukasiewicz A, Baldwin C (2017). Voice, power, and history: ensuring social justice for all stakeholders in water decision-making. Loc Environ.

[CR62] Mach KJ, Lemos MC, Meadow AM, Wyborn C, Klenk N, Arnott JC, Ardoin NM, Fieseler C, Moss RH, Nichols L, Stults M, Vaughan C, Wong-Parodi G (2020). Actionable knowledge and the art of engagement. Curr Opin Environ Sustain.

[CR63] Meadow A, Ferguson D, Guido Z, Horangic A, Owen G, Wall T (2015). Moving toward the deliberate co-production of climate science knowledge. Weather, Climate, Soc.

[CR64] Muhar A, Penker M (2018) Frameworks for transdisciplinary research: framework #5: knowledge co-production: an analytical framework. GAIA. 27(3):272. 10.14512/gaia.27.3.3

[CR65] Newcomb TJ, Simonin PW, Martinez FA, Chadderton WL, Bossenbroek JM, Cudmore B, Hoff MH, Keller RP, Ridenhour BD, Rothlisberger JD, Rutherford ES (2021). A best practices case study for scientific collaboration between researchers and managers. Fisheries.

[CR66] Norström AV, Cvitanovic C, Löf MF (2020). Principles for knowledge co-production in sustainability research. Nat Sustain.

[CR67] Patton MQ (2017). Principles focused evaluation: the guide.

[CR68] Prell C, Hubacek K, Reed M (2009). Stakeholder analysis and social network analysis in natural resource management. Soc Nat Resour.

[CR69] Prokopy L, Morton L, Arbuckle J, Mase A, Wilke A (2015). Agricultural stakeholder views on climate change: implications for conducting research and outreach. Bull Am Meteorol Soc.

[CR70] Prokopy LS, Carlton JS, Haigh T, Lemos MC, Mase AS, Widhalm M (2017). Useful to usable: developing usable climate science for agriculture. Clim Risk Manag.

[CR71] Qin Y, Abatzoglou JT, Siebert S, Huning LS, AghaKouchak A, Mankin JS, Hong C, Tong D, Davis SJ, Mueller MD (2020). Agricultural risks from changing snowmelt. Nat Clim Chang.

[CR72] Raaphorst K, Koers G, Ellen GJ, Oen A, Kalsnes B, van Well L, Koerth J, van der Brugge R (2020). Mind the gap: towards a typology of climate service usability gaps. Sustain.

[CR73] Reed MS, Curzon R (2015). Stakeholder mapping for the governance of biosecurity: a literature review. J Integr Environ Sci.

[CR74] Reed MS, Graves A, Dandy N, Posthumus H, Hubacek K, Morris J, Prell C, Quinn CH, Stringer LC (2009). Who's in and why? A typology of stakeholder analysis methods for natural resource management. J Environ Manag.

[CR75] Reed MS, Stringer LC, Fazey I, Evely AC, Kruijsen JHJ (2014). Five principles for the practice of knowledge exchange in environmental management. J Environ Manag.

[CR76] Reed MS, Vella S, Challies E, de Vente J, Frewer L, Hohenwallner-Ries D, Huber T, Neumann RK, Oughton EA, del Ceno JS, van Delden H (2018). A theory of participation: what makes stakeholder and public engagement in environmental management work?. Restor Ecol.

[CR77] Rigolot C (2020). Transdisciplinarity as a discipline and a way of being: complementarities and creative tensions. Humanit Soc Sci Commun.

[CR78] Roux DJ, Nel JL, Freitag S, Novellie P, Rosenberg E (2021). Evaluating and reflecting on coproduction of protected area management plans. Conserv Sci Pract.

[CR79] Rubin HJ, Rubin IS (2011). Qualitative interviewing: the art of hearing data.

[CR80] Singletary L, Narayanan R (2003). Assessing farmers’ willingness to participate in water banking: a case study. J Agri Ed Exten.

[CR81] Singletary L, Sterle K (2020). Supporting local adaptation through the co-production of climate information: an evaluation of collaborative research processes and outcomes. Clim Serv.

[CR82] Singletary L, Smith M, Hill G (2002). Assessing impacts on volunteers who participate in collaborative efforts to manage environmental disputes. J Volunteer Admin.

[CR83] Singletary L, Koebele E, Hockaday S, Ormerod KJ (2022). Adapting to variable water supply in the Walker River Basin.

[CR84] Singletary L, Sterle K (2018) Participatory research to assess the climate resiliency of snow-fed river dependent communities. In: Lachapelle PR, Albrecht D (eds) Addressing climate change at the community level in the United States. Community Development Research and Practice Series. Routledge, New York, pp. 53–99

[CR85] Slater K, Robinson J (2020). Social learning and transdisciplinary co-production: a social practice approach. Sustain.

[CR86] Steelman TA, Andrews E, Baines S (2019). Identifying transformational space for transdisciplinarity: using art to access the hidden third. Sustain Sci.

[CR87] Steelman T, Bogdan A, Mantyka-Pringle C (2021). Evaluating transdisciplinary research practices: insights from social network analysis. Sustain Sci.

[CR88] Tobias S, Ströbele MF, Buser T (2019). How transdisciplinary projects influence participants’ ways of thinking: a case study on future landscape development. Sustain Sci.

[CR89] Trachtenberg Z, Focht W, Sabatier PA, Focht W, Lubell M, Trachtenberg Z, Vedlitz A, Matlock M (2005). Legitimacy and watershed collaborations: the role of public participation. Swimming upstream: collaborative approaches to watershed management.

[CR90] Turnhout E, Metze T, Wyborn C, Klenk N, Louder E (2020). The politics of co-production: participation, power, and transformation. Curr Opin Environ Sustain.

[CR91] U.S. Global Climate Research Program (2018) Climate science special report: fourth national climate assessment. U.S. Global Climate Research Program, Washington, DC. https://nca2018.globalchange.gov/

[CR92] Vincent K, Daly M, Scannell C, Leathes B (2018). What can climate services learn from theory and practice of co-production?. Clim Serv.

[CR93] Vincent K, Steynor A, McClure A, Visman E, Lund Waagsaether K, Carter S, Mittal N (2021) Co-production: learning from contexts. In: Conway D, Vincent K (eds) Climate Risk in Africa: 37–56. Palgrave Macmillan, Cham. 10.1007/978-3-030-61160-6_3

[CR94] Wall TU, Meadow AM, Horganic A (2017). Developing evaluation indicators to improve the process of co-producing usable climate science. Weather Clim Soc.

[CR95] Whitton J, Carmichael A (2022) Farming in the climate emergency: socially sustainable participation in agricultural transitions. Advancing Scholarship and Practice of Stakeholder Engagement in Working Landscapes workshop series and currently under review for an international journal

[CR96] Wilds LJ (2014). Water politics in northern Nevada: a century of struggle.

[CR97] Worosz M, Stewart H, Robinette M et al. (2022) The future of farming: building a transdisciplinary team. Advancing Scholarship and Practice of Stakeholder Engagement in Working Landscapes workshop series and currently under review for an international journal

[CR98] Wyborn C, Bixler RP (2013). Collaboration and nested environmental governance: scale dependency, scale framing, and cross-scale interactions in collaborative conservation. J Environ Manage.

[CR99] Wyborn C, Datta A, Montana J, Ryan M, Leith P, Chaffin B, Miller C, van Kerkhoff L (2019). Co-producing sustainability: reordering the governance of science, policy, and practice. Annual Rev of Environ and Res.

[CR100] Zurba M, Petriello MA, Madge C (2022). Learning from knowledge co-production research and practice in the twenty-first century: global lessons and what they mean for collaborative research in Nunatsiavut. Sustain Sci.

